# Self Containment, a Property of Modular RNA Structures, Distinguishes microRNAs

**DOI:** 10.1371/journal.pcbi.1000150

**Published:** 2008-08-22

**Authors:** Miler T. Lee, Junhyong Kim

**Affiliations:** 1 Genomics and Computational Biology Program, University of Pennsylvania School of Medicine, Philadelphia, Pennsylvania, United States of America; 2 Department of Biology, University of Pennsylvania, Philadelphia, Pennsylvania, United States of America; 3 Penn Genome Frontiers Institute, University of Pennsylvania, Philadelphia, Pennsylvania, United States of America; University of Texas at Austin, United States of America

## Abstract

RNA molecules will tend to adopt a folded conformation through the pairing of bases on a single strand; the resulting so-called secondary structure is critical to the function of many types of RNA. The secondary structure of a particular substring of functional RNA may depend on its surrounding sequence. Yet, some RNAs such as microRNAs retain their specific structures during biogenesis, which involves extraction of the substructure from a larger structural context, while other functional RNAs may be composed of a fusion of independent substructures. Such observations raise the question of whether particular functional RNA substructures may be selected for invariance of secondary structure to their surrounding nucleotide context. We define the property of self containment to be the tendency for an RNA sequence to robustly adopt the same optimal secondary structure regardless of whether it exists in isolation or is a substring of a longer sequence of arbitrary nucleotide content. We measured degree of self containment using a scoring method we call the self-containment index and found that miRNA stem loops exhibit high self containment, consistent with the requirement for structural invariance imposed by the miRNA biogenesis pathway, while most other structured RNAs do not. Further analysis revealed a trend toward higher self containment among clustered and conserved miRNAs, suggesting that high self containment may be a characteristic of novel miRNAs acquiring new genomic contexts. We found that miRNAs display significantly enhanced self containment compared to other functional RNAs, but we also found a trend toward natural selection for self containment in most functional RNA classes. We suggest that self containment arises out of selection for robustness against perturbations, invariance during biogenesis, and modular composition of structural function. Analysis of self containment will be important for both annotation and design of functional RNAs. A Python implementation and Web interface to calculate the self-containment index are available at http://kim.bio.upenn.edu/software/.

## Author Summary

An RNA molecule is made up of a linear sequence of nucleotides, which form pairwise interactions that define its folded three-dimensional structure; the particular structure largely depends on the specific sequence. These base-pairing interactions are stabilizing, and the RNA will tend to fold in a particular way to maximize stability. Consider some nucleotide sequence that optimally folds into some structure in isolation; if this sequence is now embedded inside a larger sequence, then either the original structure will be a robust subcomponent of the larger folded structure, or it will be disrupted due to new interactions between the original sequence and the surrounding sequence. We explore this property of context robustness of structure and in particular define the property of “self containment” to describe intrinsic context robustness—i.e., the tendency for certain sequences to be structurally robust in many different sequence contexts. Self containment turns out to be a strong characteristic of a class of RNAs called microRNAs, whose biogenesis process depends on the maintenance of structural robustness. This finding will be useful in future efforts to characterize novel miRNAs, as well as in understanding the regulation and evolution of noncoding functional RNAs as modular units.

## Introduction

Our understanding of the significance of noncoding RNAs (ncRNAs) has increased dramatically over the last decade, notably marked by the discovery of the endogenously coded microRNAs (miRNAs) [1]–[3]. Along with the increased awareness of the diversity of ncRNAs has come a corresponding heightened attention to RNA sequence and structural measures (e.g., compared in [4]) with which to characterize known and novel RNAs.

The secondary structure of an RNA, consisting of the energy-minimizing base interactions along the length of the molecule, has a direct effect on its function [5], a fact that has been well-characterized for a variety of RNA classes. Ribosomal RNAs (rRNAs) are among the largest examples that illustrate the functional importance of RNA structure—several rRNAs along with associated proteins assemble into the large and small subunits of the ribosome, with the structural specificity to direct protein translation [6]. The cloverleaf transfer RNA (tRNA) structure allows it to associate with the ribosome and properly orient its bound amino acid during aminoacylation [7]. Various small nuclear RNAs (snRNAs) and small nucleolar RNAs (snoRNAs) are involved in RNA editing and splicing on the basis of their shape specificity [8],[9], while the stem-loop structure of precursor miRNAs (pre-miRNAs) allows them to be recognized by the ribonuclease Dicer during the miRNA maturation process [10]. Structure-derived functionality is not limited to nonprotein coding RNAs; however, some messenger RNAs (mRNAs) contain structural regulatory motifs, such as the hairpin selenocysteine insertion sequence (SECIS) that occurs predominantly in the 3′ untranslated regions (UTRs) of mRNAs coding for selenoproteins [11] and the internal ribosome entry site (IRES) in viral 5′ UTRs that promotes translation initiation in the middle of the mRNA [12]. Additionally, recognition of specific mRNAs by RNA binding proteins as well as pre-mRNA splicing all involve molecular interactions of the folded RNA structure [13],[14].

The importance of structural specificity is not limited to the end product – sequence and structural specificity during various stages of RNA biogenesis are also critical. Eukaryotic tRNAs, for example, are transcribed as longer precursor transcripts, which are recognized and cleaved on both the 5′ and 3′ ends by RNaseP and an uncharacterized endonuclease, respectively [7],[15]; some tRNAs also contain introns, which disrupt the canonical cloverleaf structure and are spliced out before the mature tRNA is exported out of the nucleus [7],[15]. The eukaryotic 18S, 5.8S, and 28S rRNAs are transcribed as a single unit and subsequently cleaved apart [16],[17]. The hammerhead ribozyme is an example of a self-splicing RNA, such that its three helices mediate cleavage of a motif that occurs on the same RNA molecule [18].

In the case of miRNAs, biogenesis begins with the transcription of long primary transcripts (pri-miRNAs), which fold into large structures that serve as substrates for the endonuclease Drosha [19]. Drosha, in complex with Pasha to form the Microprocessor complex, recognizes specific hairpin substructures in the pri-miRNA and cleaves them at the base of the helical stem region, yielding the pre-miRNA hairpins [20],[21]. These range in size from ∼60–120 nucleotides and are subsequently processed by Dicer, which targets the pre-miRNAs on the basis of their hairpin shape [22],[23]. miRNAs are notable in that the sequence of the pre-miRNA hairpin remains a robust structure through these biogenesis steps, regardless of the sequence context: when embedded in the larger primary sequence, the pre-miRNA subsequence folds into a hairpin, and when it is cleaved off to form an independent unit, the sequence folds into the same hairpin [24].

The need for context-independent structural conservation, as exemplified by the miRNA biogenesis pathway, is a hallmark of a broader phenomenon of modular composability—i.e., the generation of biopolymers through combinatorial composition of structural motifs. It is now well recognized that novel proteins can arise from shuffling of structural domains, the most vivid example being circularly permuted proteins [25],[26]. Given the critical role of structural features in RNA function and the already recognized motifs as compiled in databases such as RFAM [27], it is conceivable that many RNAs might also have arisen from evolutionary steps of domain shuffling and domain fusions. Such a process would require that the novel molecule reach a folded state that is a composition of the structural features of its parts—i.e., the structural features of the combinatorial pieces need to be invariant to composition with other sequences.

On the one hand, structural context robustness may be a product of the specific relationship between each sequence and its genomic context, a property that has been exploited in computational miRNA finders such as in [28]. On the other hand, certain subsequences may have some intrinsic tendency to be structurally indifferent to their surrounding sequence, irrespective of the particular identity of that surrounding sequence—e.g., a pre-miRNA would still be structurally robust if it were inserted into a different context. We call this property of intrinsic structural invariance “self containment.” A self-contained structural RNA (or protein) has the potential to be a modular building block in a larger structure, carry out consistent function through biochemical modifications of surrounding sequences, and potentially maintain function when inserted into novel contexts, as might occur with viral elements.

Previously, while studying the general mutational robustness of 170 structural elements of RNA viral genomes, Wagner and Stadler found that there was a trend toward higher structural robustness in conserved elements than in nonconserved elements when placed in short nongenomic contexts [29]. Using a similar approach, Ancel and Fontana studied the intrinsic context insensitivity of a set of canalized artificial RNAs, selected to have reduced environmental plasticity, and found a positive relationship between environmental canalization and modularity [30]. Other work in RNA (e.g., [31],[32]) and proteins (e.g., [33]) suggests that there is an intimate relationship between mutational robustness and domain modularity with folding kinetics, thermodynamic stability, as well as other biogenerative processes.

In this work, we analyze self containment over a broad range of biological RNAs using an intuitive scoring method to quantify different degrees of context robustness. We show that in fact pre-miRNAs do exhibit a high degree of intrinsic self containment, while most other biologically relevant RNAs tend not to show such self containment. We relate self containment to other sequence and structural features of RNA and find that no simple combination of features can completely explain self containment. Finally, we show that variation among miRNAs in degree of self containment is correlated with genomic location and miRNA-family membership, as well as their biogenerative process, as illustrated by miRNAs produced by the alternate mirtron pathway. We propose that high self containment is an intrinsic property of particular RNA sequences and may be an evolutionarily selected characteristic in molecules that need to maintain structural robustness for some aspect of their function in the face of genetic perturbations, generative perturbations, and modular composition in combinatorial contexts.

## Results

### Measuring Self Containment

Given a sequence of nucleotides *xwy*, where *w* is a sequence of interest and *x* and *y* are arbitrary upstream and downstream sequences, *w* is structurally invariant if the substructure of the *w* portion is identical to the structure of *w* in isolation. In this scenario, the paired bases in *w* are paired exclusively with other bases in *w* and do not involve the nucleotides in *x* and *y*. If *w* is structurally invariant regardless of the nucleotide identity of *x* and *y*, we call *w* self contained. We formulate self containment as a quantitative trait of *w* that varies with the degree of structural invariance vis-a-vis the pool of possible *x* and *y* sequences.

**Figure 1:**
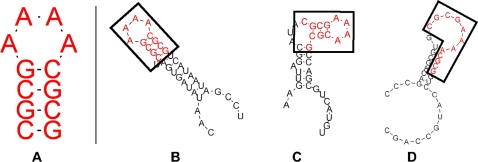
Example of varying degrees of structure preservation. (A) An RNA sequence that folds into a hairpin in isolation. (B–D) Embedding the original sequence in different surrounding sequence contexts causes varying degrees of preservation of the hairpin in the global mfe structure: complete preservation (A); loss of one base pair (B); and complete disruption of the original hairpin (C).

We developed a scoring method to measure the degree of self containment of an RNA molecule, similar to the methods used in [29] and [30] but better encapsulating the severity of structure change over a varied number of contexts. The score is calculated as follows: for each RNA sequence *w* of length *L* folding into a particular minimum free energy (mfe) secondary structure, we create a larger sequence of length 3*L* by embedding the original sequence in between randomly generated sequences *x* and *y* of equal length, forming a concatenated molecule *xwy*. We fold the resulting larger sequence and measure the proportion of the original structure preserved in the larger structure (Figure 1). We repeat the process using 1,000 different random embeddings and average the proportions to generate a single value that ranges from 0.0 to 1.0, with 1.0 indicating a maximal degree of self containment. We call this score the self-containment index (SC).

**Figure 2:**
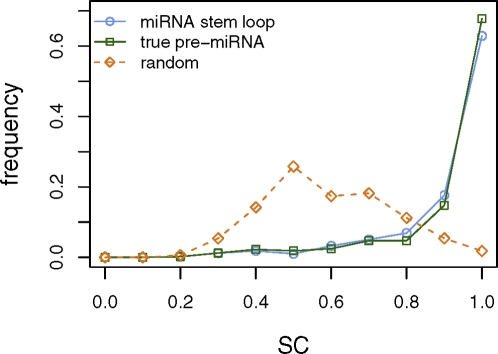
SC values for human pre-miRNA foldbacks versus random structures. Histograms of self-containment index values for the 493 human miRNA stem loops, the stem loops trimmed to represent true pre-miRNAs, and the 500 random structured RNAs.

When applied to a set of 493 human miRNA stem loops downloaded from miRBase [34],[35], filtered to exclude sequences of >90% sequence identity using the greedy sequence clustering algorithm Cd-hit [36], we found that the SC index produced a heavily right-shifted distribution, with an average SC value of 0.88 (Figure 2). We repeated the analysis on the stem-loop sequences after trimming the 5′ and 3′ ends to align with the mature miRNA sequence while including the characteristic 2-nt 3′ overhang [19],[24], thus yielding true pre-miRNA stem loops as would be produced by Drosha processing, and found the same right-shifted distribution, again with an average SC of 0.88, though true pre-miRNA SC values tend to be slightly higher than the corresponding foldback values (*p* = 0.021, Wilcoxon signed rank test) (Figure 2). In contrast, when applied to a set of 500 randomly-generated structured RNAs, generated to approximately match the length and degree of base pairing of human miRNA foldbacks (see Materials and Methods), the SC index produced a roughly normal distribution of values centered around 0.54 (Figure 2). Thus, the miRNAs exhibit a significantly higher degree of self containment than random (*p*<2.2×10^−16^, Wilcoxon rank sum test).

We tested the robustness of the SC index by varying the number of random embeddings used and found that the index gave consistent results using as few as 100 embeddings when applied to random 100-sequence subsets of the miRNA stem loops and random structures. A Pearson correlation between SC values using 100 random embeddings versus 1,000 random embeddings yielded an average slope of 0.99 with an average *r* ^2^ of 0.98, indicating that the SC index can be reliably estimated with a small sample of randomizations. (Table S1). Similarly, increasing the number of random embeddings to 5,000 also did not affect the scores (Table S1).

We also tested the effect of varying the length of the random context by comparing SC values obtained using the normal formulation—left and right random contexts of length *L*—with values obtained using context lengths ranging from 0.1*L* to 2*L*. Longer contexts produced comparable SC values to the original formulation over both miRNAs and random structures, with Pearson correlations ranging from 0.98 to 0.99 and slopes from 0.98 to 1.08. SC values were slightly but significantly lower with longer context lengths, with an average difference of 0.01 for the miRNAs and 0.04 for the random structures between the *L*- and 2*L*-derived values (*p*<1×10^−9^, Wilcoxon signed rank test). Conversely, shorter contexts produced lower correlations and inflated SC values, with the context length of 0.1*L* yielding Pearson correlations of 0.61 to 0.65 and an average increase in SC value ranging from 0.06 to 0.21 (*p*<2.2×10^−16^, Wilcoxon signed rank test) (Table S2). These data indicate that a context length of *L* is sufficient to model the effects of large sequence surroundings, but lengths much shorter than *L* may be insufficient.

Finally, we tested the degree to which the base composition of the random contexts affected the SC values and found that substituting random contexts with coding sequence, intronic sequence, or versions of these with shuffled dinucleotides (i.e., the nucleotide sequences were randomly permuted in a way that preserves both the mononucleotide and dinucleotide frequencies of the original [37],[38]) had little effect on SC values. Pearson correlations between SC values produced by the original formulation compared to each of these variants, for each of the RNA classes, yielded slopes ranging from 0.91 to 1.08 with *r* ^2^ values from 0.86 to 0.98 (Table S3), again suggesting that the SC index can be well estimated using randomization experiments.

### RNA Classes Have Varying Degrees of Self Containment

**Figure 3:**
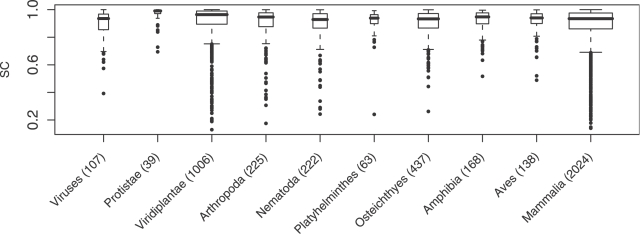
SC values for pre-miRNAs from various lineages. Box-and-whisker plots showing the self-containment index distribution among pre-miRNAs found in miRBase, indicating the median in bold, the interquartile range enclosed by the box, the smallest and largest non-outliers indicated by the whiskers, and outliers represented as individual points. The lineages displayed are, from left to right: viruses; protists; plants; and animals divided into the phyla arthropods, nematodes, flatworms, and chordates, which are further subdivided into classes/superclasses of fish, amphibians, birds, and mammals. Number of miRNAs for each lineage is shown in parentheses, and box width is proportional to the square root of this number.

Using the SC index, we analyzed several other RNA classes to characterize their degrees of self containment. First, to confirm that high self containment is not particular to miRNAs in humans, we measured the self containment of the miRNA stem loops spanning the 56 other species represented in miRBase [34],[35]. We found that among species with at least five annotated miRNAs in miRBase, the average SC was between 0.85 and 0.98 (Table S4), and that the distributions of scores when grouped into larger taxonomic classes were all heavily right shifted, as was the case for the human miRNAs (Figure 3).

**Figure 4:**
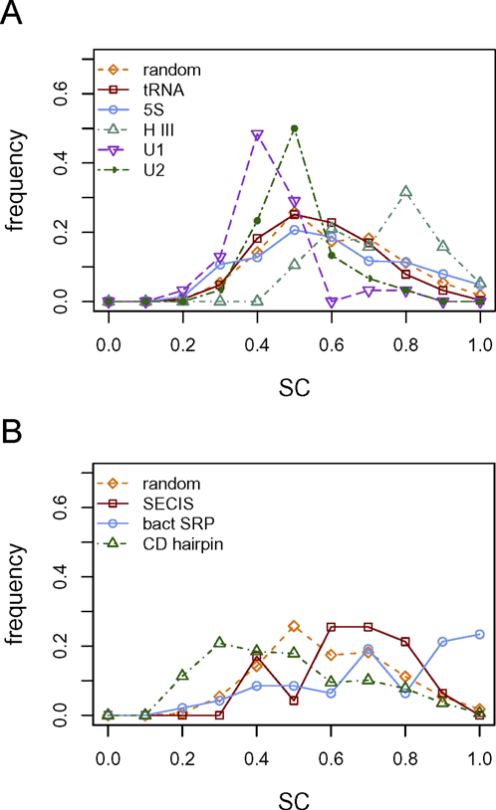
SC values for RNA classes. Histograms of self-containment index values for (A) tRNAs, 5S rRNAs (5S), Hammerhead type III ribozymes (H III), U1 spliceosomal RNAs, and U2 spliceosomal RNAs, as compared to random structures; and (B) SECIS elements, bacterial SRP RNAs (bact SRP), and hairpins derived from protein-coding regions of mRNAs (CD hairpin), as compared to random structures.

We next measured the self containment of several other classes of structural RNAs that have been compared previously using other measures, e.g., [4],[37],[39]: tRNAs, U1 and U2 spliceosomal RNAs, Hammerhead type III ribozymes, and 5S rRNAs (Table 1). All of these yielded SC ranges much lower than for the miRNAs (Figure 4A). The Hammerhead III ribozymes exhibited the highest average degree of self containment at 0.69, which is significantly lower than for the miRNAs (*p* = 3.95×10^−8^, Wilcoxon rank sum test), while the remaining classes had average SC values ranging from 0.38 for U1 to 0.54 for the 5S rRNA (Figure 4A).

**Table 1:** Average self-containment index values for RNA classes analyzed.

RNA class	Number of sequences	Average SC
miRNA (all species)	4,429	0.90
miRNA (human)	493	0.88
Hammerhead III ribozyme	19	0.69
Bacterial SRP	47	0.69
RFAM-extracted hairpins	9,572	0.65
SECIS elements	47	0.60
5S rRNA	290	0.54
Random structures	500	0.54
tRNA	751	0.51
U2 spliceosomal	30	0.46
CD hairpins	168	0.43
U1 spliceosomal	31	0.38

To determine whether high self containment is a product of a strong hairpin shape, which these other classes lack, we additionally analyzed selenocysteine insertion sequences (SECIS) and bacterial signal recognition particle (SRP) RNAs from RFAM [27], both of which exhibit strong hairpin secondary structures. We also tested a set of hairpins derived from the protein-coding regions of mRNA transcripts, originally curated to serve as a negative training set for pre-miRNA detection (CD hairpins) [40]. Both the SECIS and SRP RNAs exhibited higher SC values than all the other structural RNAs except for the Hammerhead ribozymes, yielding average values of 0.60 and 0.69, respectively; however, this was still significantly lower than for the miRNAs (*p* = 2.2×10^−16^ for SECIS, *p* = 7.24×10^−12^ for SRP, Wilcoxon rank sum test) (Figure 4B). The CD hairpins, despite their structural similarity to pre-miRNAs, turned out to have very low self containment, with an average SC value of 0.43, greater only than that of the U1 RNAs (Figure 4B, Table 1).

### Two Additional Groups of Hairpins Show High Self Containment

In a further attempt to find groups of RNAs with similar SC distributions to the miRNAs, we considered the entire set of RFAM sequences [27],[41], filtered to >90% sequence identity. We extracted all unbranched hairpins greater than 50 nucleotides in length, with at least half of the nucleotides involved in base pairs; these hairpins were either full-length RNAs, or they were structurally decomposable portions of full RNAs. In all, we obtained 9,572 hairpins, of which 335 were miRNAs.

**Figure 5:**
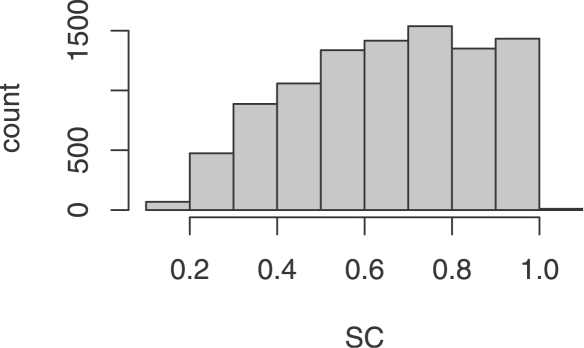
SC values for RFAM-extracted hairpins. Histogram of self-containment index values for the 9,572 hairpins extracted from RNAs annotated in RFAM.

We computed SC values for each hairpin. As a whole, there exists a bias toward higher SC values, though the distribution is roughly uniform among the SC values greater than 0.5 (Figure 5). We extracted the top 15% scoring hairpins, which corresponds to having a SC value greater than 0.900, and looked for overrepresentation of hairpins deriving from particular RFAM families. Nineteen classes show significant enrichment with *p*<0.001 according to a Fisher's exact test, of which 12 are miRNA families (Table 2). Of the remaining classes, the eukaryotic SRP RNA and the hepatitis C virus stem-loop VII show the most significant skews toward high self containment, with the majority of the individuals having SC values greater than 0.9, as was observed among the miRNA stem loops. The next most significant non-miRNA class are hairpins derived from U2, which do not show as pronounced a skew.

**Table 2:** RFAM families whose hairpin structures are significantly enriched for high self containment.

Class	Total number of hairpins	Observed in top 15% SC	Expected by chance	*p*-value^a^
MIR (combined)^b^	335	285	50.3	3.70×10^−82^
RF00017 SRP_euk_arch	171	105	25.7	9.76×10^−25^
RF00468 HCV_SLVII	41	31	6.2	4.11×10^−10^
RF00451 mir-395	31	27	4.7	6.32×10^−10^
RF00075 mir-166	21	20	3.2	3.74×10^−8^
RF00445 mir-399	17	16	2.6	9.49×10^−7^
RF00073 mir-156	15	15	2.3	1.26×10^−6^
RF00004 U2	113	43	17.0	1.93×10^−6^
RF00169 SRP_bact	110	37	16.5	7.17×10^−5^
RF00247 mir-160	10	10	1.5	7.70×10^−5^
RF00074 mir-29	9	9	1.4	1.78×10^−4^
RF00238 ctRNA_pND324	10	9	1.5	2.98×10^−4^
RF00103 mir-1	10	9	1.5	2.98×10^−4^
RF00551 bicoid_3	19	12	2.9	3.15×10^−4^
RF00256 mir-196	13	10	2.0	3.28×10^−4^
RF00027 let-7	13	10	2.0	3.28×10^−4^
RF00053 mir-7	8	8	1.2	4.12×10^−4^
RF00047 mir-2	8	8	1.2	4.12×10^−4^
RF00042 CopA	12	9	1.8	7.41×10^−4^
RF00244 mir-26	7	7	1.1	9.62×10^−4^

a By Fisher's exact test.

b All miRNA families combined.

### Self-Containment Index Correlates with Other RNA Measures

Having characterized the extent to which self containment varies among different RNAs, we next sought to understand the biophysical basis of SC by comparing it to other measures on structured RNAs. We compared SC values with 14 other measures drawn from [4] and [39]: sequence length; %GC nucleotide content; mfe and mfe normalized by length [4],[42] and GC content [42],[43]; normalized Shannon entropy of base-pair probabilities among all the structures in the thermodynamic ensemble (Q) [44]; base-pairing proportion overall (P) and the proportion of those pairs that are AU, GC, and GU pairs; *z*-scores of mfe, Q, and R when compared to a set of shuffled sequences preserving dinucleotide frequencies [37],[38]; and the stability of the mfe structure with respect to competing alternate structures, which is approximated by the number of structures in the thermodynamic ensemble within 2 kcal/mol of the mfe [31],[45] (see Materials and Methods). To test whether self containment is related to the complexity of an RNA sequence, we also compared SC to the Shannon entropy of nucleotide, dinucleotide, and trinucleotide probabilities across the sequence. Finally, we tested whether self containment depends more on the strength of base interactions in the 5′ and 3′ ends of the sequence rather than in the interior of the structure, using the base-pairing proportion measure limited to the distal portions of the sequence (see Materials and Methods).

**Figure 6:**
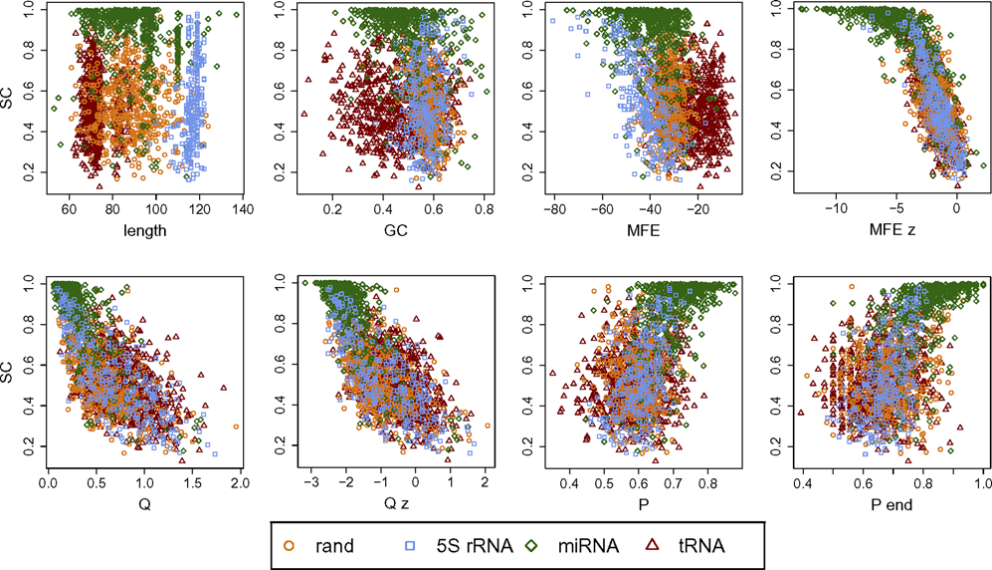
Comparison of SC with other RNA measures. Scatter plots showing self-containment index plotted against eight other RNA measures: sequence length (length); proportion of G and C nucleotides (GC); minimum free energy of the structure (MFE); *z*-score of the mfe compared to 1,000 dinucleotide-shuffled sequences (MFE *z*); normalized Shannon entropy of base-pair probabilities among all the structures in the thermodynamic ensemble (Q); *z*-score of Q compared to 1,000 dinucleotide-shuffled sequences (Q *z*); proportion of bases involved in base pairs over the entire structure (P); and proportion of bases involved in base pairs, limited to the 5′ and 3′ ends of the sequence. Four sets of RNAs are overlaid in each plot: tRNAs, random structures, 5S rRNAs, and human pre-miRNAs.

We used four RNA classes for comparison: human miRNA stem loops, random structured RNAs, 5S rRNAs, and tRNAs. The correlations between variance-stabilized SC values—using an arcsin square-root transform (see Materials and Methods)—and values obtained from each of these measures are presented in Table 3, and scatter plots for length, GC content, mfe, mfe *z*-score, Q, Q *z*-score, P, and end-restricted P are presented in Figure 6.

**Table 3:** Correlation coefficients (*r* ^2^) between self-containment index and other RNA measures.

Measure	miRNA	Random	5S rRNA	tRNA
Length	0.04	0.01^b^	0.12	0.00^b^
GC proportion^a^	0.15	0.18	0.02	0.01^b^
mfe	0.07	0.00^b^	0.46	0.04
Length-normalized mfe	0.21	0.03	0.44	0.05
GC-normalized mfe	0.31	0.06	0.63	0.27
mfe *z*-score	0.58	0.35	0.72	0.48
Base pair entropy (Q)	0.56	0.35	0.59	0.25
Base pair entropy *z*-score	0.58	0.37	0.56	0.28
Base pair proportion (P)^a^	0.25	0.00^b^	0.26	0.01
Base pair proportion *z*-score	0.30	0.04	0.29	0.05
AU base pair proportion^a^	0.21	0.14	0.00^b^	0.01
GC base pair proportion^a^	0.13	0.06	0.03	0.00
GU base pair proportion^a^	0.05	0.02	0.03	0.09
End base pair proportion^a^	0.33	0.04	0.27	0.01
End AU base pair proportion^a^	0.16	0.08	0.00^b^	0.01
End GC base pair proportion^a^	0.09	0.02	0.02	0.00^b^
End GU base pair proportion^a^	0.03	0.03	0.03	0.09
Number of alternate structures	0.12	0.04	0.14	0.09
Nucleotide entropy	0.02	0.12	0.02	0.00^b^
Dinucleotide entropy	0.01	0.05	0.01^b^	0.01
Trinucleotide entropy	0.00^b^	0.01^b^	0.00^b^	0.01

a Proportion metrics were variance stabilized by performing an arcsin-square root transform before correlation was calculated.

b Correlation was not significant (*p*>0.05).

For many of these measures, the relationship with SC varies depending on the class of RNA considered. Minimum free energy, for example, is moderately correlated with SC in the 5S rRNAs, but this is not the case for the other classes. Similarly, base-pairing proportion—overall, partitioned into base-pair type, or limited to particular regions of the structure—is moderately predictive for miRNAs and 5S, but not for tRNAs. Sequence complexity, as described by the nucleotide entropy measures, appears to have little to no relationship on self containment. The strongest correlations are between SC and mfe *z*-score, as well as with base pair entropy and the corresponding *z*-score, which themselves have all been shown to have strong correlations with one another [4].

We performed a multiple regression using all 21 variables, to assess how SC relates to a linear combination of the various RNA measures. The linear model yielded an *r* ^2^ of 0.52 for the random structures, 0.65 for tRNAs, 0.76 for miRNAs, and 0.81 for the 5S rRNAs. However, the significantly predictive variables for the regression model differed between the RNA classes, suggesting that self containment reflects a subtler sequence-structure relationship that is not captured in a common model across these factors and RNA classes.

### RNA Sequences Have Enhanced Self Containment Given Their Structure

To further characterize the relationship between structure and sequence in determining degree of self containment, we generated an ensemble of 100 inverse-folded sequences for each human miRNA stem loop using RNAinverse from the Vienna RNA Package [46]; each inverse-folded sequence is predicted to adopt the respective miRNA structure with minimum free energy. We then measured self containment for each set of sequences to produce a distribution of SC values for each miRNA structure and compared these distributions.

**Figure 7:**
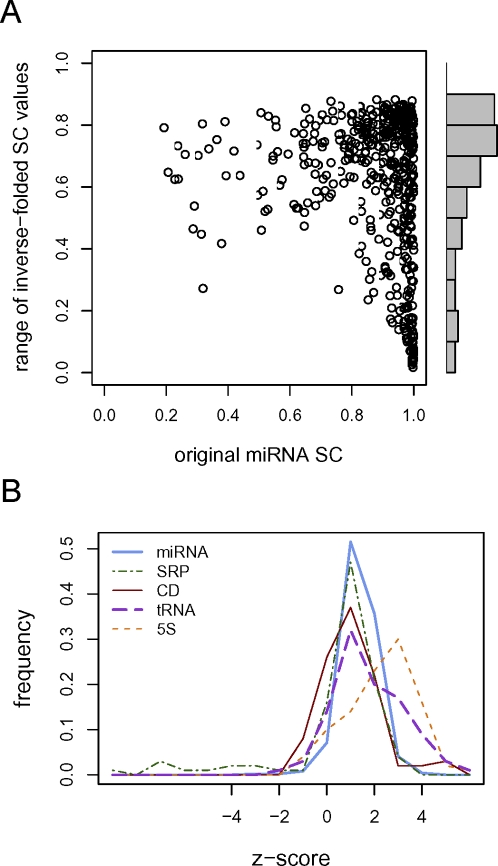
SC values of RNAs versus inverse-folded sequences. (A) Scatter plot showing self-containment index values for each original pre-miRNA versus the range of SC values observed among 100 inverse-folded sequences with the same structure as that miRNA. A range value of 0 indicates homogeneity among the SC values obtained over all 100 inverse-folded sequences, while higher values indicate higher diversity. The marginal histogram of range values is also shown. (B) Histograms showing the RNA class distributions of *z*-scores calculated from the self-containment index values of each RNA compared to the SC values of its 100 inverse-folded sequence ensemble. Classes shown are human pre-miRNAs (miRNA), hairpins derived from protein-coding transcripts (CD), hairpins derived from eukaryotic signal recognition particle RNAs (SRP), 5S rRNAs (5S), and tRNAs.

Some of the structures have very narrow ranges of admissible SC values, particularly on the high end where it appears that there are structures that are context-robust regardless of the sequence. However, most of the structures admit a wide range of possible SC values, even among structures whose real miRNA sequences exhibit very high self containment, indicating that self containment is not simply determined by structure but is an evolved feature of the sequence given a particular structure (Figure 7A). The same trend was observed when other types of RNA were considered (data not shown).

Using the ensemble of 100 inverse-folded sequences per miRNA stem-loop structure, we calculated the average SC value and standard deviation and compared this to the SC value of the true miRNA sequence by computing a *z*-score. We found a strong tendency for the real sequences to have higher self containment than average, though few of them had *z*-scores greater than 2 (Figure 7B). We performed the same analysis on random 100-sequence subsets of the 5S rRNAs, tRNAs, CD hairpins, and the eukaryotic SRP RNA-derived hairpins we previously extracted, and found that all classes displayed right-shifted *z*-score distributions, indicating that the biological RNA sequences tend to be more self contained than artificial sequences that fold into the same structure (Figure 7B).

### Mirtrons Are Less Self Contained Than Conventionally Processed miRNAs

The high self containment that distinguishes miRNAs is hypothesized to be partly a function of their unique biogenesis mechanism; therefore, we tested whether enhanced self containment would still be present in the absence of the biogenesis constraint. Recently, several intronic miRNAs were characterized in *Drosophila melanogaster* [47],[48] and *Caenorhabditis elegans* [48] that bypass the Drosha cleavage pathway. Instead, these “mirtrons” are full-length intronic sequences that are spliced from protein-coding transcripts through the normal splicing pathway, giving rise to pre-miRNA foldbacks that are subsequently processed by Dicer to yield mature miRNAs. Since mirtrons are processed as introns, structural robustness of the hairpin shape is not as critical to biogenesis as it is for pre-miRNAs that need to be excised by Drosha. We hypothesized that this effect would be reflected in lower SC values for mirtrons as compared to canonical pre-miRNAs.

For the mirtrons identified in *Drosophila* [47],[48], this does appear to be the case. We compared the SC values of the 14 mirtrons *dme-mir-1003–1016* against the remaining 76 *Drosophila* miRNAs (filtered to exclude sequences >90% similar) and found that mirtrons have lower SC values on average—0.83 for mirtrons versus 0.91 for canonical miRNAs; this difference achieves a significance level of *p* = 0.062 according to a *t* test on logit-transformed SC values. An additional degenerate *Drosophila* mirtron was characterized, *dme-mir-1017*, that is aligned to only the 5′ splice site and has a long 3′ overhang, which presumably is cleaved subsequent to intron splicing [48]. Including *dme-mir-1017* in the analysis, after trimming the sequence from the 3′ end to yield a canonical hairpin, achieves a 5% significance level (*p* = 0.0483) (Table 4).

**Table 4:** Average self-containment index differences between mirtrons and canonical pre-miRNAs.

Species	Number of mirtrons	Average mirtron SC	Average miRNA SC	*p*-value^a^
*D. melanogaster*	15	0.83	0.91	0.0483
*C. elegans*	4	0.98	0.88	7.06×10^−3^
*H. sapiens*	13	0.50	0.88	4.96×10^−6^
*M. mulatta*	11	0.67	0.89	2.39×10^−5^

a By a Wilcoxon rank sum text (*H. sapiens*) or by a *t* test (all others).

Among mammalian mirtrons that have recently been characterized [49], the effect is much stronger. Thirteen human and 11 *Macaque mulatta* mirtrons were identified with strong cloning evidence and sequence conservation, including one previously annotated miRNA, *mir-877*. When we compared SC values between the human mirtrons and the set of canonical miRNA stem loops excluding *hsa-mir-877*, we found that human mirtrons had an average SC of 0.50 compared to the canonical 0.88 with *p* = 4.96×10^−6^, using a Wilcoxon rank sum test due to the non-normality of the data (Table 4). Similarly, macaque mirtrons also had a significantly lower average SC of 0.67, compared to 0.89 for the canonical miRNAs (*p* = 2.39×10^−5^, *t* test) (Table 4).

In contrast, this trend was not observed in *C. elegans*—all four of the mirtrons identified in *C. elegans* [48] were found to be more highly self-contained than the average *C. elegans* miRNA (*p* = 7.06×10^−3^, *t* test) (Table 4). Since mirtrons in different lineages may not have a common ancestry [49], perhaps this trend reflects a different biogenesis mechanism or evolutionary history.

### Self Containment Distinguishes miRNA Subclasses

Although high self containment seems to be a distinguishing characteristic for Drosha-processed miRNAs, there is still variability in the degrees of self containment among these miRNAs. We sought to account for some of this variability by measuring mean differences in SC along several functional partitions of the set of human miRNAs.

Among the full set of 533 unfiltered human miRNAs, we tested the tendency for self containment to differ among miRNAs depending on their family membership. The miRNAs belonging to a miRNA family as annotated in miRBase [34],[35]—i.e., possessing at least one ortholog or paralog—were found to be significantly more self contained, with an average SC of 0.91, than the nonconserved miRNAs, which had an average SC of 0.78 (*p* = 1.32×10^−7^, Wilcoxon rank sum test) (Table 5). This significance is possibly inflated by the fact that, by definition, miRNAs in a family share nucleotide sequence, which would cause some correlation in SC values among individuals in the same family. Using a more stringent formulation, obtained by averaging the human SC values per family and performing a rank sum test on family averages versus the SC values of the nonconserved miRNAs, we were still able to see the significant difference (*p* = 1.37×10^−4^). Additionally, we confirmed the result by performing a randomization test (see Materials and Methods), which is robust to sampling bias and distribution shape (*p*<10^−5^). Restricting the analysis to only the miRNAs with human paralogs, we again found a significantly higher degree of self containment when compared to the human miRNAs lacking human relatives (*p* = 1.05×10^−4^, Wilcoxon rank sum test; *p*<10^−5^, randomization test).

**Table 5:** Average self-containment index differences across different human pre-miRNA groups.

miRNA group	In group count	In group average SC	Out of group count	Out of group average SC	*p*-value^b^
In miRNA family	404	0.91	129	0.78	1.00×10^−5^
In human miRNA family^a^	251	0.92	282	0.84	1.00×10^−5^
Intergenic	225	0.91	303	0.86	1.54×10^−3^
Exon overlapping	53	0.81	475	0.89	7.69×10^−3^
Clustered	241	0.91	287	0.86	1.20×10^−4^

a Belonging to a miRNA family with multiple human members.

b By a randomization *t* test (see Materials and Methods).

A large proportion of human miRNAs occur in genomic clusters [50] as part of the same primary transcript [24],[51],[52]. Using a liberal definition of clustering proposed by [28], such that a miRNA is part of a cluster if it is <10,000 nucleotides from another miRNA on the same strand, we found that miRNAs occurring in clusters are significantly more self contained than isolated miRNAs (*p* = 1.48×10^−4^, Wilcoxon rank test) (Table 5). Since clustering turns out to be correlated with family membership (*p*<2.2×10^−16^, *χ* ^2^ test, 1 degree of freedom), we again used a randomization test to confirm significance (*p* = 1.2×10^−4^).

Finally, we tested whether miRNAs overlapping genes had differing self containment than intergenic miRNAs. Using miRBase annotations [34],[35], miRNAs classified as intergenic were significantly more self contained than gene-overlapping miRNAs (*p* = 0.0195, Wilcoxon rank sum test) (Table 5). When broken down into intron- versus exon-overlapping miRNAs, the effect is stronger, with exon-overlapping miRNAs significantly less self contained than non-exon-overlapping miRNAs (*p* = 1.5×10^−4^, Wilcoxon rank sum test). Again, among human miRNAs there is an association between family membership and genomic location—intergenic miRNAs are overrepresented in families (*p* = 2.86×10^−10^, *χ*2 test, 1 degree of freedom) and exon-overlapping miRNAs are underrepresented in families (*p* = 4.84×10^−3^, *χ* ^2^ test, 1 degree of freedom). Randomization tests again confirmed significance of the SC differences (*p* = 1.54×10^−3^ for intergenic versus gene-overlapping, *p* = 7.69×10^−3^ for exon-overlapping versus non-exon-overlapping).

## Discussion

In the previous sections we showed that there exist RNA sequences that have an intrinsic tendency to maintain their specific folded structure regardless of their embedded sequence context. We developed a way to measure this tendency, the self-containment index, and we used the index to show that degree of self containment varies among functional classes of RNA. miRNAs, with their need to maintain structural invariance through two cleavage steps during biogenesis, exhibit an enhanced degree of self containment, in contrast to other classes of RNAs without such a restriction. When we considered a subset of miRNAs, mirtrons, that bypass one of these cleavage steps, we found a significantly lower average self containment in three species. Among human miRNAs, we found a positive association of high self containment with membership in human-specific or cross-species miRNA families and putative transcription in a polycistronic cluster; as well as with location of the miRNAs in genomic regions not overlapping protein-coding genes. We postulate that self containment is potentially an evolved feature of particular RNA classes rather than a characteristic purely determined by the physicochemical characteristics of folded RNA.

It is possible that possessing some degree of self containment is simply an inherent property of biological RNAs. For example, small RNA subsequences that are also thermodynamically stable may be fast-folding in the kinetic folding pathway (P. Higgs, Personal Communication). Such elements would obtain their base pairing first, which would inhibit their interaction with larger sequence elements. Thus, a certain degree of self containment may be posited to be an epiphenomenon of the folding kinetics. We did observe a strong relationship between SC and other measures that typically denote structurally relevant RNAs, particularly measures for structural saturation (base pair proportion), sequence-conditional structural stability (mfe *z*-score), and structural specificity (base-pair entropy) (Table 3). And, the fact that biological RNA sequences appear to have enhanced self containment given their structure (Figure 7B) reflects this trend as well. However, the extreme degree of self containment exhibited by the miRNAs and not by many other similarly shaped and stable RNAs seems to suggest that there is functional relevance to self containment that goes beyond being just a byproduct of structural relevance. And, as pointed out in Hartling and Kim [33] as well as Ancel and Fontana [30], there may be an inherent coupling between the modularity of biopolymer structures and both the equilibrium distribution and kinetic pathways of the folding process. Thus, selection for self containment may be mediated through fast-folding and vice versa.

The decreased self containment of mirtrons as compared to miRNAs that are processed by Drosha (Table 4) is evidence that the structural requirements of miRNA biogenesis at least partly explain the tendency toward high self containment. The current model for mirtron biogenesis suggests that mirtrons are spliced from mRNAs as conventional introns, with the formation of a lariat structure covalently linking the 5′ splice junction with the 3′ branch point, effectively isolating the mirtron sequence from the surrounding exonic sequence; it is only after splicing and subsequent debranching that the characteristic pre-miRNA hairpin shape is fully realized [47],[48]. Thus, mirtrons do not need to be “presented” as a context-insensitive substructure the way canonical miRNA hairpins are in the context of the primary transcript. As a result, mirtrons may be more free to accumulate nucleotide changes that lead to lower self containment, provided that the final spliced hairpin structure is not affected, whereas changes in a canonical pre-miRNA might affect recognition by Drosha due to structure disruption in the context of the primary transcript. Or, a novel proto-mirtron with lower self containment might more easily enter the miRNA processing pathway than a corresponding proto-canonical miRNA, which would additionally have to be structurally compatible with its surrounding sequence.

Still, the biogenesis mechanism may not provide sufficient a priori reason why pre-miRNAs should exhibit high *intrinsic* structural robustness, as opposed to structural invariance given their specific genomic contexts. Perhaps the ability to remain robust over many different genomic contexts reflects an explicit mechanism to buffer against change. At the local level, genomic instability of the surrounding primary transcript would be unlikely to affect the structure of a highly self-contained precursor stem loop, and hence would be less likely to disrupt Drosha recognition. Primary transcript sequence immediately surrounding the stem-loop sequence has been shown to be poorly conserved [50],[53], suggesting that miRNA precursor sequences do experience a high degree of instability of surrounding sequence. On a more global scale, high self containment would allow for reinsertion of a pre-existing miRNA or a copy into a novel genomic context, again with a high probability that the embedded stem-loop structure would be preserved. The trend for conserved and clustered miRNAs to exhibit higher self containment (Table 5) supports the idea that functional miRNAs arising from genomic modifications such as duplications and rearrangements [54] were better buffered against context change and thus were maintained. Conversely, a miRNA with low self containment would be less likely to give rise to functional paralogs—the duplicated sequence would tend not to fold correctly in the new context, making preservation of the duplicate miRNA sequence less likely due to significant loss of function.

If high self containment allows miRNA stem loops to be modular units, potentially able to function in different genomic contexts, then we might ask why selection for modularity would exist for miRNAs. In fact, the organization of miRNAs into primary polycistronic transcripts would seem to be facilitated by modularity of the stem loops, especially given that there are several clusters that contain ostensibly unrelated miRNAs [50] that may have resulted from several insertion events. The role of the primary transcript appears to be to facilitate the expression of several miRNAs at once [24], which would allow easy neofunctionalization of a duplicated miRNA if it is inserted into a primary transcript under different regulation from the source miRNA. But we might also imagine a situation where the release of individual pre-miRNAs from the primary transcript can be modulated, perhaps through RNA binding elements that block access by Drosha. This suggests a model of the primary transcript as a way to organize functionally related miRNAs while simultaneously allowing for fine-tuned control of their individual activities. Furthermore, if miRNA hairpins can be easily inserted or moved around, we can then envision the primary transcript as a collection of miRNA building blocks that can be combined and swapped over evolutionary time according to the evolving regulatory needs of the cell, a mechanism that would be difficult to attain if miRNAs were not as highly self contained.

The high self containment of miRNAs is also interesting given that they have additional sequence constraints that are ostensibly unrelated to the hairpin structure. Among miRNAs that overlap functional regions of another gene, we observed a significant decrease in average self containment (Table 5), indicating that these miRNAs are not as free to evolve high self containment, since any nucleotide changes leading to higher self containment might adversely affect the function of the overlapping gene. miRNAs are also constrained to maintain target specificity – loss of complementarity of the mature sequence with the target inhibits miRNA-driven regulation [55], so in a sense, miRNA hairpins are not as freely able to evolve toward highly self-contained sequences, unless compensatory changes occur in the target sequence as well. However, given that the majority of miRNAs do have high self containment, it is also possible that there are constraints on the space of possible target sequences, such that some classes of sequences are disfavored as targets if the resulting complementary miRNA hairpins would all have low self containment. Further work is necessary to determine whether this is a quantifiable effect that can be exploited for target prediction.

As a strong indicator for miRNAs, the property of self containment can be used in future computational miRNA search strategies, as evidenced by the ability of SC to discriminate between pre-miRNAs and pseudo-hairpins (Figure 4B, Table 1), which have been repeatedly used as negative training data for miRNA prediction (e.g., [40],[56],[57]). For de novo design applications, ensuring high self containment among candidate structures would serve as an effective filter for hairpins that can be robustly inserted into different genetic contexts.

Beyond its potential role in miRNAs, self containment is to a certain degree a requisite property of biopolymers that form through combinatorial elaboration of modular parts. A functional fusion biopolymer cannot be generated if the fused sequences do not retain their original substructures. Recently, Rigoutsos et al. [58] have described the existence of an extensive collection of repeated RNA elements in the human genome that have combinatorial arrangements, potentially suggesting that combinatorial generation might be an important feature of novel RNA elements. We propose that understanding the self-containment properties of RNAs and their structural components is fundamental to understanding the extent to which RNAs are modular molecules, such that large RNAs can be decomposed into a set of structurally robust building blocks that can potentially be swapped out or rearranged.

## Materials and Methods

### Software and Implementation

We used the default settings of the standalone RNAfold and RNAinverse programs bundled in the Vienna RNA Secondary Structure Package [46] for RNA secondary structure prediction and inverse folding respectively. We used a Python implementation of the Altschul-Erikson algorithm [59] for dinucleotide shuffling written by P. Clote [60]. All other code was custom written using Python and run on Linux machines. High-volume computation, including calculating SC and other structural measures on RNAs, was performed using approximately 40–60 nodes of a Linux cluster. Sequence filtering to exclude highly similar sequences was done using Cd-hit, which implements a greedy clustering algorithm [36]. RNA structure drawings were produced using RNAViz [61]. Graphs were produced using R [62].

### RNA Sequence Sets

All miRNA foldback sequences were obtained from miRBase release 10.0 [34],[35]. To obtain the “true” pre-miRNA set, we trimmed these sequences according to the structure annotation found on miRBase such that the hairpin was truncated on the 5′ end to align with the mature sequence in the case of 5′-derived mature miRNAs or the miR* sequence in the case of 3′-derived mature miRNAs; and similarly truncated on the 3′ end, creating a 2-nt 3′ overhang (Dataset S1). CD hairpin sequences were obtained from [40]. All other RNA sequences were obtained from RFAM 8.0 seed and full sequence lists [27],[41]. Any wildcard IUPAC nucleotide characters found in the RFAM sequences were replaced with a random consistent RNA nucleotide (e.g., “B” would be replaced with either “C,” “G,” or “U” with equal probability).

Random RNA sequences were generated to approximately match the statistics of human miRNA foldbacks. For each candidate sequence, a random length was chosen from a normal distribution with mean 89 and standard deviation 12.6 (the approximate average length and standard deviation of human miRNA foldbacks), and an RNA sequence was generated using uniform nucleotide probabilities; sequences shorter than 61 or longer than 137 nucleotides (again based on human miRNA shortest and longest lengths) were discarded. Candidates were folded using RNAfold, and only candidates with mfe values within one standard deviation of the average mfe for a miRNA foldback of that length were retained. The resulting set of 500 random sequences had an average length of 88.9 bases and an average minimum free energy of −32.8 kcal/mol (Dataset S1).

Genomic coordinates, gene overlap, and family membership for the human miRNAs were also obtained from miRBase [34],[35]. Of the 533 human miRNAs in the database, five lacked genomic location information (*hsa-mir-672*, *hsa-mir-674*, *hsa-mir-871*, *hsa-mir-872*, and *hsa-mir-941-4*) and were thus left out of any analysis that depended on these features.

### Calculating the Self-Containment Index

For each sequence of interest *w* with length *L*, a set of 2*n* random sequences of length *L* are generated, where *n* is a user-defined parameter determining the number of random contexts to test—typically 1,000. The sequence *w* is folded using RNAfold and the structure stored in Vienna RNA parenthesis-dot notation, struct(*w*). For each pair of random sequences *x* and *y*, a concatenated sequence *xwy* is created and folded using RNAfold, then the portion of the Vienna structure corresponding to the index positions of *w* is extracted, struct′(*w*). struct′(*w*) is modified to create a legal RNA structure by replacing inconsistent parentheses (indicating bases paired with bases outside of *w*) with dots (indicating unpaired bases). Hamming distance is calculated between struct(*w*) and struct′(*w*) and divided by *L*, and the resulting proportion is subtracted from 1 to obtain *p_i_* for the *i*th random context. All of the *p_i_*'s are averaged to obtain the final self-containment index value.

Self-containment index values for all RNAs analyzed are listed in Dataset S2.

For the runs using biological sequence contexts rather than random contexts, we generated a set of one thousand coding and intronic segments from randomly selected human NCBI Reference Sequence genes [63] downloaded from the UCSC Genome Bioinformatics Site [64]. Segments were extracted from a random interval at least 20 nucleotides from either end of the spliced transcript sequence for the coding sequence, or of the concatenated introns with any repetitive sequence removed using RepeatMasker [65] for the intronic sequence. Dinucleotide-shuffled sets were created from these sets as well.

### RFAM Hairpin Extraction

We started with the entire RFAM full RNA set and filtered it using Cd-hit to exclude 90% similar sequences, resulting in 26,239 sequences. We folded all of the sequences using RNAfold, then extracted all hairpin substructures. We discarded all substructures of length less than 50 nucleotides, substructures where fewer than half the bases were involved in base pairs, and any hairpins with branching, defined in terms of the Vienna representation as containing a left parenthesis in the string to the right of the first right parenthesis. We calculated SC on the resulting set of 9,572 hairpins, using *n* = 100 random contexts. The hairpin sequences are included in Dataset S1, while the SC values are included in Dataset S2.

### RNA Sequence and Structural Measures

All measures were calculated based on previous descriptions (e.g., [4],[39]). Base pairing entropy (Q) was calculated using the formulation in [44]. End base pairing proportion was calculated by summing the number of paired bases contained in the first (5′) one-fourth and the last (3′) one-fourth of the sequence and dividing by half the sequence length. Sequence entropies were calculated using single base probabilities (i.e., the number of A, C, G, and U bases occurring in the sequence each divided by the length of the sequence) in the Shannon entropy equation *H* = −∑ *p_i_* log2(*p_i_*) for the mononucleotide case; using probabilities of each of the possible 16 consecutive nucleotide combinations (e.g., AA, AC, …, UU) in the dinucleotide case; and using the 64 three-consecutive nucleotide combinations in the trinucleotide case.

We reimplemented the algorithm described in [31] to characterize the number of alternate suboptimal structures of a sequence. For each sequence, all suboptimal structures within 2 kcal/mol of the mfe were obtained using RNAsubopt in the Vienna RNA Package. We filtered the results and kept only local minimum structures, defined to be structures such that removal or addition of a single base pair increases the global free energy.

Correlations were calculated using arcsin-square-root (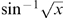) transformed values for the proportion measures such as SC (i.e., with values on [0,1]) to normalize the variances—the arcsin transformation spreads out values near 0 and 1, reducing the impact of low variance at these boundaries on the statistical analysis [66]. Values from non-proportion measures were used directly.

### Statistical Tests

For the randomization tests, we randomly shuffled the assignment of arcsin-square-root transformed SC values to labels (miRNA names, belonging to group A versus group B) *N* = 100,000 times and calculated a two-sided p-value as the number of times the absolute *t* statistic was greater than the original absolute *t* statistic, divided by *N*. We used the Welch *t* statistic for unequal sample variances, 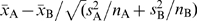 where *x̅*_A_ is the average of the group A values, 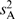 the sample A variance, and *n*_A_ the number of members in group A; and similarly for group B.

For parametric hypothesis testing, SC values were logit transformed (ln(*x*/1−*x*)) to normalize the data – similar to the arcsin transform, the logit transform spreads out values near 0 and 1, though in a more extreme manner to shape the data to assume a more normal-like distribution [66]. Normality was verified using the Shapiro-Wilk test, and similarity of variance was assessed using an F test. Mean differences were tested using a two-sample, two-sided independent *t* test, with null hypothesis that the mean difference is 0. Data that did not exhibit normality were subjected to a two-sided Wilcoxon rank sum test, or signed rank test if paired.

### Availability

A Python implementation of the self-containment index calculation, as well as a web interface for direct sequence queries, will be made available at http://kim.bio.upenn.edu/software/.

## Supporting Information

Table S1 Effects of varying the number of random contexts used to calculate the self-containment index.
(0.01 MB PDF)

Table S2 Effects of varying the length of the random contexts used to calculate the self-containment index.
(0.01 MB PDF)

Table S3 Effects of varying the source of the random contexts used to calculate the self-containment index.
(0.01 MB PDF)

Table S4 Average self-containment index values for non-human miRNAs.
(0.01 MB PDF)

Dataset S1 RNA sequences generated.
(1.2 MB TXT)

Dataset S2 Self-containment index values for RNAs.
(1.2 MB)
