# Supplementary material for: Self Containment, a Property of Modular RNA Structures, Distinguishes microRNAs
Source: PLoS Comput Biol. 2008 Aug 22;4(8):e1000150. doi: 10.1371/journal.pcbi.1000150 (PMC2517099; doi:10.1371/journal.pcbi.1000150)
Supplement: Table S1 — Effects of varying the number of random contexts used to calculate the self-containment index. (0.01 MB PDF) [file pcbi.1000150.s001.pdf]

Table S1. Effects of Varying the Number of Random Contexts Used to Calculate the Self-containment Index

# Contexts Used	RNA	Slope[a]	r^2 [b]
100	miRNA	1.00	0.98
	rand	0.97	0.98
5000	miRNA	1.00	1.00
	rand	0.99	0.99

[a]Slope of the linear regression line for the modified score as a function of the normal formulation of SC (using 1000 random contexts).

[b]Correlation coefficient between the modified score and the normal formulation of SC.
