# Supplementary material for: Self Containment, a Property of Modular RNA Structures, Distinguishes microRNAs
Source: PLoS Comput Biol. 2008 Aug 22;4(8):e1000150. doi: 10.1371/journal.pcbi.1000150 (PMC2517099; doi:10.1371/journal.pcbi.1000150)
Supplement: Table S2 — Effects of varying the length of the random contexts used to calculate the self-containment index. (0.01 MB PDF) [file pcbi.1000150.s002.pdf]

Table S2. Effects of Varying the Length of the Random Contexts Used to Calculate the Self-containment Index

Length of Context[a]	RNA	Slope[b]	r^2 [c]	Avg. SC Difference[d]	p-value[e]
0.1L	miRNA	0.46	0.65	0.06	2.20E-16
	rand	0.78	0.61	0.21	2.20E-16
0.5L	miRNA	0.90	0.99	0.01	2.22E-16
	rand	0.98	0.97	0.05	2.20E-16
1.5L	miRNA	1.06	0.99	-0.01	9.76E-10
	rand	0.98	0.99	-0.02	2.20E-16
2L	miRNA	1.08	0.99	-0.01	6.55E-15
	rand	0.99	0.98	-0.04	2.20E-16

[a]Length of the random context appended to each end of the query sequence, with respect to L, the length of the query sequence. Under the normal formulation of SC, the context length is equal to L.

[b]Slope of the linear regression line for the modified score as a function of the normal formulation of SC (using a context length of L).

[c]Correlation coefficient between the modified score and the normal formulation of SC.

[d]Difference between the average SC value obtained using contexts of length L and the average SC value using the modified length context.

[e]By a Wilcoxon signed rank test.
