# Supplementary material for: Self Containment, a Property of Modular RNA Structures, Distinguishes microRNAs
Source: PLoS Comput Biol. 2008 Aug 22;4(8):e1000150. doi: 10.1371/journal.pcbi.1000150 (PMC2517099; doi:10.1371/journal.pcbi.1000150)
Supplement: Table S3 — Effects of varying the source of the random contexts used to calculate the self-containment index. (0.01 MB PDF) [file pcbi.1000150.s003.pdf]

Table S3. Effects of Varying the Source of the Random Contexts Used to Calculate the Self-containment Index

context source	RNA	slope[a]	r^2 [b]
coding sequence	miRNA	1.04	0.98
	rand	1.02	0.98
intron	miRNA	0.92	0.97
	rand	0.99	0.96
shuffled coding	miRNA	1.01	0.98
	rand	1.01	0.98
shuffled intron	miRNA	0.89	0.96
	rand	0.99	0.96

[a]Slope of the linear regression line for the modified score as a function of the normal formulation of SC (using random contexts).

[b]Correlation coefficient between the modified score and the normal formulation of SC.
