# Supplementary material for: Self Containment, a Property of Modular RNA Structures, Distinguishes microRNAs
Source: PLoS Comput Biol. 2008 Aug 22;4(8):e1000150. doi: 10.1371/journal.pcbi.1000150 (PMC2517099; doi:10.1371/journal.pcbi.1000150)
Supplement: Table S4 — Average self-containment index values for non-human miRNAs. (0.01 MB PDF) [file pcbi.1000150.s004.pdf]

Table S4. Average Self-containment Index Values for Non-Human miRNAs

Abbrev.	Species	Clade[a]	n[b]	Average SC
xla	Xenopus laevis	Amphibia	7	0.86
xtr	Xenopus tropicalis	Amphibia	177	0.92
aga	Anopheles gambiae	Arthropoda	38	0.89
ame	Apis mellifera	Arthropoda	54	0.89
bmo	Bombyx mori	Arthropoda	20	0.93
dme	Drosophila melanogaster	Arthropoda	93	0.89
dps	Drosophila pseudoobscura	Arthropoda	26	0.91
gga	Gallus gallus	Aves	154	0.91
age	Ateles geoffroyi	Mammalia	45	0.90
bta	Bos taurus	Mammalia	105	0.91
cfa	Canis familiaris	Mammalia	5	0.90
cgr	Cricetulus griseus	Mammalia	1	0.98
ggo	Gorilla gorilla	Mammalia	86	0.88
lla	Lagothrix lagotricha	Mammalia	48	0.88
lca	Lemur catta	Mammalia	16	0.86
mml	Macaca mulatta	Mammalia	71	0.89
mne	Macaca nemestrina	Mammalia	75	0.90
mdo	Monodelphis domestica	Mammalia	100	0.91
mmu	Mus musculus	Mammalia	432	0.87
oar	Ovis aries	Mammalia	3	0.74
ppa	Pan paniscus	Mammalia	89	0.89
ptr	Pan troglodytes	Mammalia	83	0.89
ppy	Pongo pygmaeus	Mammalia	84	0.89
rno	Rattus norvegicus	Mammalia	290	0.90
sla	Saguinus labiatus	Mammalia	42	0.88
ssc	Sus scrofa	Mammalia	53	0.92
cbr	Caenorhabditis briggsae	Nematoda	90	0.90
cel	Caenorhabditis elegans	Nematoda	134	0.88
dre	Danio rerio	Osteichthyes	337	0.89
fru	Fugu rubripes	Osteichthyes	131	0.93
tni	Tetraodon nigroviridis	Osteichthyes	78	0.92
sme	Schmidtea mediterranea	Platyhelminthes	63	0.91
cre	Chlamydomonas reinhardtii	Protistae	39	0.96
ath	Arabidopsis thaliana	Viridiplantae	174	0.92
bna	Brassica napus	Viridiplantae	3	0.99
gma	Glycine max	Viridiplantae	21	0.91
mtr	Medicago truncatula	Viridiplantae	17	0.98
osa	Oryza sativa	Viridiplantae	189	0.94
ppt	Physcomitrella patens	Viridiplantae	211	0.86

pta	Pinus taeda	Viridiplantae	22	0.93
ptc	Populus trichocarpa	Viridiplantae	151	0.92
sof	Saccharum officinarum	Viridiplantae	8	0.94
smo	Selaginella moellendorffii	Viridiplantae	54	0.96
sbi	Sorghum bicolor	Viridiplantae	60	0.94
tae	Triticum aestivum	Viridiplantae	29	0.85
zma	Zea mays	Viridiplantae	79	0.95
ebv	Epstein Barr virus	Viruses	22	0.89
hsv	Herpes Simplex Virus 1	Viruses	2	0.94
hcm	Human cytomegalovirus	Viruses	11	0.91
hiv	Human immunodeficiency virus 1	Viruses	2	0.48
ksh	Kaposi sarcoma-assoc. herpesvirus	Viruses	12	0.87
mdv	Mareks disease virus	Viruses	25	0.89
mgh	Mouse gammaherpesvirus 68	Viruses	9	0.91
rlc	Rhesus lymphocryptovirus	Viruses	16	0.92
rrv	Rhesus monkey rhadinovirus	Viruses	7	0.96
sv4	Simian virus 40	Viruses	1	0.90

[a]Taxonomic group. For the metazoa, species are divided into phyla, with the vertebrates further subdivided into classes.

[b]Number of miRBase-annotated miRNAs for the species, after filtering to remove sequences with >90% similarity.
